# An assessment of the relevance of the home neighbourhood for understanding environmental influences on physical activity: how far from home do people roam?

**DOI:** 10.1186/s12966-015-0260-y

**Published:** 2015-08-16

**Authors:** Melvyn Hillsdon, Emma Coombes, Pippa Griew, Andy Jones

**Affiliations:** College of Life and Environmental Studies, Exeter University, Richards Building, St Lukes, Heavitree Road, Exeter, EX1 2LU UK; Norwich Medical School, University of East Anglia, Norwich, NR4 7TJ UK; UKCRC Centre for Diet and Activity Research (CEDAR), Box 296, Institute of Public Health, Forvie Street, Robinson Way, Cambridge, CB2 0SR UK

**Keywords:** Physical activity, Environment, Neighbourhood, Accelerometer, Global Positioning Systems

## Abstract

**Background:**

The choice of geographical unit of analysis in studies of the built environment and physical activity has typically been restricted to the home neighbourhood where only a small proportion of physical activity may actually be undertaken. This study aimed to examine the distance from home at which physical activity takes place and how this varies by personal and neighbourhood characteristics.

**Methods:**

A cross-sectional, population based study of 195 people in the North West region of England, aged 18 to 91 years, clustered in 60 localities (small geographical areas of ~125 households). Individual socio-demographic data were collected by computer-aided personal interviews and physical activity was characterised by accelerometer and Global Positioning System (GPS) data. The locations of periods of light, moderate and vigorous intensity physical activity (LMVPA) undertaken outdoors were linked to measures of the neighbourhood around the home and distance from home.

**Results:**

Sixty per cent of outdoors LMVPA took place outside of the proximal home neighbourhood (800 m buffer). Distances from home where median levels of LMVPA were undertaken varied by gender (*p* < 0.05), home location, area deprivation, and car ownership (all *p* < 0.001).

**Conclusions:**

Objectively measured physical activity appears to vary appreciably by participant characteristics and home location, although for many settings a large proportion is undertaken outside of the home neighbourhood, suggesting the characterisation of neighbourhoods close to home will fail to properly capture the environmental influences on physical activity.

## Background

Reversing the alarming downward trend in levels of physical activity in the UK and elsewhere is a key stage in addressing many of the current pressing public health concerns regarding chronic disease and mental wellbeing [1]. In recent years research attention has particularly focused on the role of characteristics of the built environment, both as a reason for the observed declines in activity but also as a potential solution [2]. It is hypothesized that the built environment will be associated with physical activity because it influences opportunities for recreation, active transport and domestic activities such as gardening. It can also provide safe and pleasant routes to everyday destinations. Yet systematic reviews have reported only modest relationships and some counterintuitive findings [3, 4].

The lack of observed associations between the built environment and physical activity behaviours may partly result from the fact that many studies focus on the characteristics of residential neighbourhoods close to home. Study participants are typically grouped into administrative units or some other form of area delineation and the features of these are examined (e.g. [5–7]). Alternatively, the characteristics of a zone around the home of each participant, typically 1-2 km in radius, are studied (e.g. [8–10]). Yet a potentially important limitation of these techniques is that many people may undertake a substantial proportion of their physical activity in other settings some distance from home.

In recent work questioning the relevance of typically employed neighbourhood definitions, Jones and colleagues found no evidence that 11 year old children living in the same neighbourhood, defined by groupings of administrative units, showed similar levels of physical activity in Bristol, England [11]. Further, based on a mapping exercise, Villanueva and colleagues found that 800 m and 1600 m neighbourhood buffers around home did not correspond well to actual activity spaces used by 10–12 year old Australian children [12]. Amongst adults, it is reasonable to believe that a focus on the area proximal to home is even less appropriate than that for children, who will have less independent mobility [13]. Indeed a recent GPS based study by Prins and colleagues found that in Dutch older adults, half of walking trips had a distance from home of over 720 m and for cycling trips this distance was over 1.6 km, demonstrating that many adult’s day-to-day active travel trips extend beyond the zone that researchers would traditionally consider as the home neighbourhood [14].

As a consequence of these findings, studies are now seeking to identify the wider activity spaces that individuals make use of for physical activity. Participant’s activity spaces have been defined using a range of methods, including asking participants to delineate on a map the space they make use of on a day-to-day basis (e.g. the VERITAS tool described by Chaix and colleagues [15]) and by using travel diaries to record the places that people commonly visit (e.g. [16]). Global Positioning Systems (GPS) are also beginning to be used to provide new insights into environments used [17] as they allow individual’s locations to be continuously monitored and, when used in conjunction with accelerometers, they provide objective data about both the level and location of physical activity performed [18, 19]. Their application thus means it is now possible to test whether the equivocal results from studies using neighbourhood based measures might arise because much activity is not taking place within the neighbourhood definitions commonly employed.

A small number of recent studies have combined objectively measured physical activity with GPS data in adults and reported the amount of physical activity undertaken inside and outside of the home neighbourhood. A cross-sectional study of 148 US adults reported that less than 20 % of recorded MVPA took place within a 1 km buffer of home [20], whilst a second study of 41 US adults reported that around 50 % of MVPA took place within 833 m of home and 45.6 % greater than 1.7 km from home [21]. In a pilot study of 35 US adults, 54 % of MVPA was undertaken outside of the participants’ neighbourhood (a 1.54 km buffer) [18]. However, a limitation of these works is that none of them have examined how the distance from home that activity is undertaken varies according to characteristics of the home location such as urban/rural status or the characteristics of the sample such as age, gender etc. There is evidence from self-report for example that rural adults may make less use of their neighbourhoods for recreational walking than their urban counterparts [22]. Further, a mixed-method study of activity space size amongst a sample of rural residents of Northern Ireland found that low-income individuals tended to roam less far from home than affluent residents due to financial constraints and poor public transport provision acting to force them to participate in activities closer to home or on main transport corridors [23]. It is important to understand that studies may be limited in their ability to detect associations between physical activity and environmental characteristics if the scale of the environment being measured is inappropriate and raises the possibility that environmental measurements may need to be tailored to the particular characteristics of study participants and the settings within which studies are undertaken.

This study builds on the existing literature using accelerometer and GPS data collected amongst a diverse sample of English adults to examine the distance from home at which physical activity is undertaken and investigate how this varies by gender, age, home location, area deprivation, and car ownership.

## Methods

### Setting

The Forty Area STudy (FAST) gathered data on the physical activity patterns of a sample of men and women aged 18+ living in private households in the North West region of England. The initial primary sampling unit was forty census Output Areas (OA; geographical areas standardised for population size, geographical shape, dwelling type and housing tenure) [24], which were selected from all possible OAs in the region. The number was extended to 60 following an interruption to recruitment due to snow. During the interruption, we reviewed recruitment rates and decided to increase the number of sampling units to increase the final sample size. A stratified random sample was used for the selection of OAs to ensure maximal sample variance with regard to an eight category urban–rural measure [25], which was then collapsed into three categories that included ‘urban’ (population >10,000), ‘town and fringe’ (population between 1,500 to 10,000), and ‘rural’ (population <1,500). The sample also additionally ensured maximal variance with regard to tertiles of the English Indices of Multiple Deprivation (IMD), which is a single deprivation score covering a range of economic, social and housing issues [26]. All data were collected between September 2010 and May 2011.

### Participants

A random sample of 48 postal addresses from each OA was selected using the UK PostCode Address File [27]. Each address was sent an advance letter about the study prior to a home visit by a trained interviewer. One adult per household was selected for interview. The total sample included 1084 participants representing a 50.7 % recruitment rate. All participants were asked to wear an accelerometer for 7 days, whilst a random sub-sample (25 %, *n* = 265) were additionally asked to wear a GPS device. Of the participants who were asked to wear a GPS device, 16 were excluded from analysis because they provided no data and a further 54 because they failed to provide at least one day of ten hours of accelerometer wear time. This left 195 people who met the accelerometer wear time requirements and these individuals form the sample for the present analysis.

### Measures

Each participant completed a computer assisted personal interview. Elicitation questions were those used in the annual National Health Survey for England [28]. Individual socio-demographic information collected pertinent to this analysis were gender, age, and whether the household of the respondent owned a car. Home locations were identified using the Ordnance Survey Address Layer database based on home postcodes (zip codes). In the UK a single postcode corresponds to an average of 15 addresses and in our study area postcode zones typically ranged from between 0.009 and 0.829 km^2^ in size for urban and rural postcodes respectively. Participants were assigned an area based deprivation measure based on the IMD score for the OA within which their home residence fell, and urban–rural status was similarly ascertained based on residential OA.

To provide an objective measure of physical activity, participants wore an accelerometer device (Actigraph GT1M; Actigraph LLC, FL, USA). This was set to record at a 60 s epoch, and participants were asked to wear it on a supplied belt around their waist during waking hours for 7 days following interview. On the same belt they also wore a GPS (Qstarz BT-Q1000XT; Taipei, Taiwan) which recorded a location (latitude and longitude) every 5 s. Because neither device was waterproof, participants were instructed to remove the belt when in water.

### Neighbourhood delineation

The boundaries of the home neighbourhood for each participant were objectively characterised using the ArcGIS 9.2 Geographical Information System (GIS) (ESRI Inc, Redlands, California). In order to provide a comparison with commonly employed neighbourhood definitions, the area surrounding the location of each participant’s home postcode was defined as the area within 800 m (approximately corresponding with a 10 min walk) along the pedestrian network (roads plus footpaths) of each address.

### Data preparation and analytical methods

Software was written in Java to match accelerometry data points to the closest recorded GPS location based on their date and time-stamps. The accelerometer recorded at a 1 min interval whilst the GPS recorded at a 5 s interval. To match the timestamps of the two devices without losing any of the GPS data points, we first reproduced the accelerometer data at a 5 s interval by repeating each count per minute observation 12 times, and then matched the GPS location onto each of these. After the accelerometer and GPS data were matched, data points that had a time difference of ≤30 s between the accelerometer timestamp and that of its matched GPS location were considered valid for inclusion in the study. Matched data points with a time difference greater than this, for example where the GPS was switched off or had lost signal, were considered as missing locational information because the participant might have moved to a new unrecorded location. The analysis we present does not therefore include the accelerometer data associated with these instances. Further, we removed any GPS data points where there was a change in location that resulted in a speed measurement of ≥90mph as these were most likely associated with poor GPS location fix. In addition, any GPS data points and associated accelerometer data that were recorded within 30 s of the device being switched on were also excluded as the location fix is unstable during this warming-up period, and so the accuracy of the locations that the GPS device records are poorer [29].

Accelerometer derived measures of physical activity were calculated for valid days (at least 10 h wear time excluding periods of continuous zeros of >60 min). As it was not possible to differentiate times when the GPS had no signal due to being indoors from times when it was not being worn and switched off, no minimum wear time requirements were set for the GPS. Based on recorded counts per minute (CPM), each accelerometer data point was then classified as light (500–2019 CPM), moderate (2020–5999 CPM) or vigorous intensity activity (≥6000 CPM) [30] from which total time spent per day in light, moderate and vigorous physical activity combined (LMVPA) was computed. We focused on LMVPA rather than solely higher intensity activity to ensure that we captured time spent walking. To maximise the available data, participants with at least 1 day of valid wear time for the accelerometer were included for analysis (*n* = 195).

GPS devices do not perform well inside buildings and often fail to acquire a satellite signal meaning that the wearer’s location is either not recorded at all or is recorded but with a low degree of precision. Consequently this analysis focuses only on physical activity recorded outdoors and any GPS data points that were missing or appeared to be recorded inside a building were excluded from this work. In order to identify these indoor points, the processed data points were entered into the GIS package ArcGIS 9.2 and overlaid with a digital map of building outlines that was generated from the Ordnance Survey MasterMap dataset [31]. Spatial queries were then undertaken to identify the location of outdoor data points that were at LMVPA intensity, and were overlaid with the neighbourhood boundaries around the home of each participant to identify points falling inside and outside the neighbourhood. In addition, the straight line distance from home was calculated for each point.

The percentage of total recorded LMVPA for participants was calculated according to whether points were inside or outside the home neighbourhood and differences in percentages by gender, age, urban–rural status, area deprivation (above or below the median score), and car ownership were examined using Mann–Whitney U and Kruskal Wallis H tests. The median distance from home at which LMPVA was recorded for participants was also calculated, and again differences in median distances by personal characteristics and home location were examined using Mann–Whitney U and Kruskal Wallis H tests.

## Results

The characteristics of the 195 sample participants are detailed in Table 1. When compared with those 70 study participants who did not meet the inclusion criteria, the included sample were slightly more active, undertaking 13.4 min of LMVPA per hour of device wear time overall compared to 10.7 min for those excluded (*p* = 0.037) and were more likely to own a car (83.1 % vs 71.4 %, *p* = 0.038). There were no other statistically significant differences.

**Table 1:** Characteristics of the included sample (*n* = 195). Values are either the number of participants and column percent or the median value and interquartile range (IQR)

	Urban	Town & fringe	Rural	Total
	(*n* = 133)	(*n* = 23)	(*n* = 39)	(*n* = 195)
Personal characteristics				
Gender: male (*n*, %)	55 (41.4)	8 (34.8)	19 (48.7)	82 (42.1)
Age group:				
18–39 years (*n*, %)	41 (30.8)	5 (21.7)	9 (23.1)	55 (28.2)
40–64 years (*n*, %)	63 (47.4)	9 (39.1)	22 (56.4)	94 (48.2)
65+ years (*n*, %)	29 (21.8)	9 (39.1)	8 (20.5)	46 (23.6)
IMD score (range: 3.9-78.7, high is more deprived) (median, IQR)	31.6 (21.2)	22.9 (50.2)	12.0 (8.6)	23.7 (29.9)
Car ownership: ‘yes’ (n, %)	108 (81.2)	17 (73.9)	37 (94.9)	162 (83.1)
Device wear time				
Device wear time: total hours per person recorded during the study including time spent indoors (median, IQR)	70.9 (22.4)	71.1 (16.6)	75.1 (18.4)	71.3 (19.8)
Physical activity				
LMVPA: total hours per person recorded during the study including time spent indoors (median, IQR)	15.3 (8.5)	15.2 (11.7)	13.7 (11.7)	15.3 (9.3)
Mins of LMVPA per hour of device weartime per person (median, IQR)	13.8 (7.2)	12.7 (9.4)	13.0 (6.3)	13.4 (7.1)

Abbreviations: *IMD* Indices of Multiple Deprivation, *LMVPA* light, moderate and vigorous intensity physical activity

For the included sample, those living in rural areas were more likely to have access to a car (95.4 % of participants) versus 73.1 % in urban areas and 84.3 % in town and fringe locations. Furthermore all of our rural participants were classified as living in an affluent area, compared to 37.1 % for urban participants and 66.7 % for town and fringe.

Compliance with device wear was excellent with participants providing a median of 71.3 h (interquartile range 19.8 h) of matched data across the 7 study days (Table 1). Overall we had a median of 15.3 h (interquartile range 9.3 h) of LMVPA data per person across the 7 study days, and after dropping indoor data this left a median of 16.4 mins of outdoors LMVPA per person per day (interquartile range 29.6 mins), confirming that people spend the majority of their time indoors [32].

**Fig. 1:**
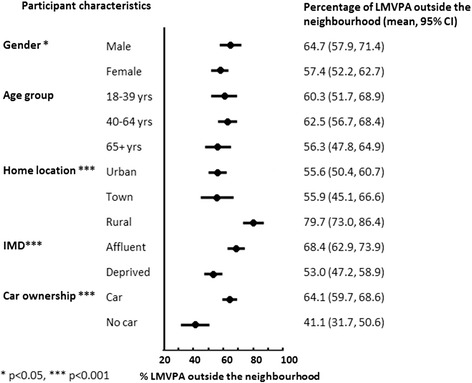
Percentage of recorded outdoor LMVPA falling outside the home neighbourhood according to personal and neighbourhood characteristics

**Fig. 2:**
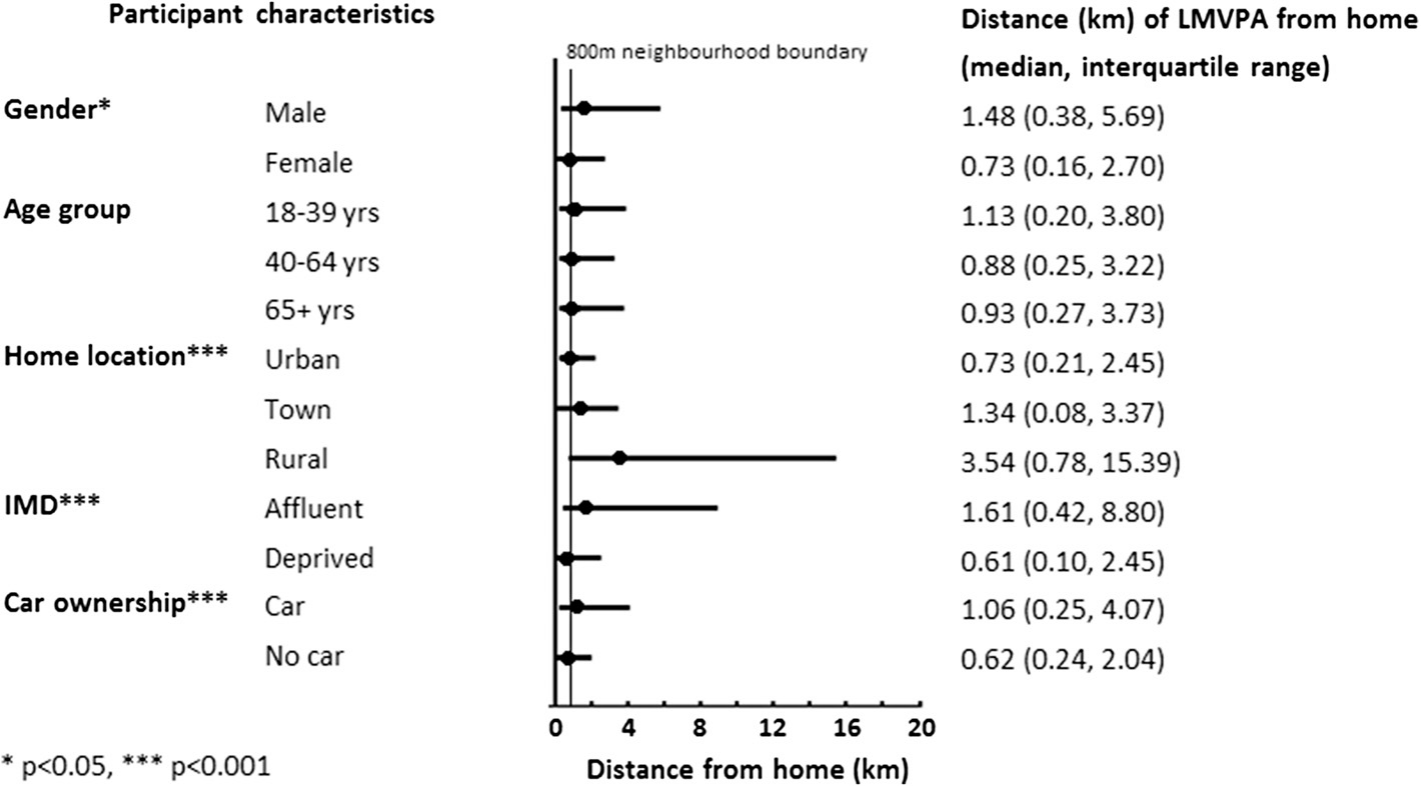
Median (and interquartile range) distance from home where outdoor LMVPA takes place by personal and neighbourhood characteristics

Figure 1 shows that, overall, 60.5 % of outdoor LMVPA was undertaken outside the home neighbourhood, as defined by the area within 800 m of the home. Males, participants residing in rural areas and those in more affluent areas undertook a higher proportion of their LMVPA outside the home neighbourhood compared with other groups, but there was little difference between respondents according to their age. As anticipated, car owners also undertook more of their LMVPA outside the home neighbourhood. The median distance from home where LMVPA took place was associated with gender (*p* < 0.05), home neighbourhood location, area deprivation, and car ownership (all at *p* < 0.001) as shown in Fig. 2. As expected, differences in distance were in the same direction as those observed for comparisons of LMVPA inside and outside the neighbourhood.

## Discussion

This study provides new insights into the physical activity patterns of English adults with respect to where activity is undertaken. In particular, we found that when the home neighbourhood was delineated as the area within a 10 min walk (800 m) from home, more LMVPA was actually undertaken outside the boundaries rather than within them. The distance from home at which LMVPA took place varied by setting, being more local in urban settings, but appreciably further away in rural areas, towns and the urban fringe. In urban areas the median distance just fell within our 800 m neighbourhood definition, but for residents of the other areas it was well outside. Variations in distance were also associated with gender, area deprivation and car access.

Our findings show clearly that the delineation of neighbourhoods based on a 10 min walk from home, or similar distance based buffer designed to represent the home neighbourhood, will poorly capture the relevant environments people use for physical activity. Importantly, we have also shown that the distance from home by which activity takes place will vary according to participant characteristics and study setting. In accordance with the results of Troped and colleagues [20], we found that males tended to undertake a greater proportion of their activity further from home. We also found that rural location of residence, high area affluence, and car ownership were associated with a statistically significant greater proportion of LMVPA being performed outside the typically defined home neighbourhood as well as a higher median distance from home for activity. In some cases disparities were substantial. For example, rural participants undertook 80 % of their LMVPA outside the neighbourhood compared to just 56 % for their urban counterparts. These findings suggest that delineations of home neighbourhoods based on simple distance based criteria are unlikely to capture the full extent of built environment influences on physical activity, and further that the appropriate scale of measurement may differ according to study and participant characteristics. We propose that only using the built environment attributes of the proximal home neighbourhood is one of the key reasons for the equivocal nature of the evidence base to date, and that the relevant geographical scale will vary by environmental exposure, type of physical activity, type of residential location and population subgroups.

Rather than a-priori selecting single neighbourhood buffers for local area characterization, we propose future studies should use more flexible geographical scales that reflect the physical activity spaces of each person and take account of the other contextual factors mentioned above. GPS and GIS provide the opportunity to objectively measure the location and spatial scale at which different types of physical activity take place, although the logistical overheads of employing GPS amongst large population samples are considerable, and GPS data may not be available for many previously collected datasets. Where GPS is unavailable, researchers should consider varying the size of buffer according to the urban–rural setting, the characteristics of each study participant, and the types of activity being studied. The median distances we present in Fig. 2 provide some guide to potentially suitable buffer sizes, although further work is required to test the sensitivity of buffer definition to observed associations between environmental characteristics and physical activity.

This study’s strengths include that the sample size was larger than other similar studies [18, 20, 21]. It consisted of 73.6 % of the total number of participants in FAST who were asked to wear a GPS device and produced valid data, which is better than many studies. Furthermore the sample included adults from a wide range of heterogeneous area types including urban, town, and rural areas across a whole region. Also, both environments and behaviours were objectively measured reducing the possibility of misclassification.

In terms of limitations, we were only able to measure the location of physical activities undertaken outdoors. Many participants will have spent long periods indoors, for example while at work or in the home, and since the GPS is unlikely to have held a satellite signal for all of these times it was not possible to exclude participants from analyses according to the number of hours of valid GPS data they provided. As a consequence we may have excluded some physical activity from our analysis that was motivated by facilities present outside the neighbourhood. For example, individuals may travel outside their neighbourhood to visit a sports centre and whilst our analysis would capture physical activity associated with the journey to this facility, we have not considered physical activity undertaken indoors.

A further limitation is that although we attempted to remove indoor data by excluding GPS points that fell inside a building there is likely to have been some misclassification of the land parcel that each data point fell within. This is due to the imprecision in some recorded GPS points associated with use of the devices in locations where tall buildings are present and obscure the satellite signal the device uses to ascertain location [33]. The included sample under represents people living in deprived, rural areas and therefore it is possible that their patterns of physical activity differ from those reported. In addition, the number of people in non-urban areas overall was small and therefore they may be unrepresented. However, the sampling strategy was designed to produce a sample representative of the region of the country from which it was drawn.

The results that we present are for light, moderate and vigorous physical activity combined and it may be that different patterns could be observed for different forms or intensities of physical activity. For example, some activities such as gardening may be more likely to be undertaken in the home neighbourhood than others such as participation in sports. Although we did not have information on the specific activities undertaken by our participants we did test the sensitivity of our findings by repeating our analyses for moderate and vigorous activity intensities only. The results we obtained were substantively extremely similar to those presented here suggesting that activity context may not have a strong impact on our findings. We are not, however, able to comment on the generalisability of our results to countries with sustainably different climates to the UK, where individuals might spend different amounts of time outdoors due to cultural or climatic conditions. Finally, we used 60-s epochs for the accelerometer data to maximise battery life and to focus on purposeful activity. Consequently, we may have underestimated the total volume of LMVPA undertaken, but we do not believe this would substantially alter our findings.

## Conclusions

This study indicates that adults undertake most of their outdoor light, moderate and vigorous physical activity outside their proximal home neighbourhood. The spatial scale relevant to physical activity varies according to home neighbourhood location, area deprivation, demographic factors and the environmental exposure being examined. Studies that are limited to a single context close to home are likely to underestimate the true relationship between physical activity and the built environment.
